# Clade 2.3.2 Avian Influenza Virus (H5N1), Qinghai Lake Region, China, 2009–2010

**DOI:** 10.3201/eid1703.100948

**Published:** 2011-03

**Authors:** Xudong Hu, Di Liu, Mingyang Wang, Le Yang, Ming Wang, Qingyu Zhu, Laixing Li, George F. Gao

**Affiliations:** Author affiliations: China Agricultural University College of Veterinary Medicine, Beijing, People’s Republic of China (X. Hu, Ming Wang, G.F. Gao);; Chinese Academy of Sciences (CAS) Key Laboratory of Pathogenic Microbiology and Immunology Institute of Microbiology, Beijing (X. Hu, D. Liu, Mingyang Wang, G.F. Gao);; Graduate University CAS, Beijing (Mingyang Wang, G.F. Gao);; Northwestern Institute of Plateau Biology CAS, Xining, China (L. Yang, L. Li);; Tibet Plateau Institute of Biology, Lhasa, Tibet Autonomous Region, China (L. Yang);; State Key Laboratory of Pathogens and Biosecurity Academy of Military Medical Sciences, Beijing (Q. Zhu);; Beijing Institutes of Life Sciences CAS, Beijing (G.F. Gao)

**Keywords:** Avian influenza virus, influenza, viruses, H5N1, clade 2.3.2, Qinghai Lake, China, letter

**To the Editor:** In 2005, a large population of wild migratory birds was infected with highly pathogenic avian influenza (HPAI) virus (H5N1) in the Qinghai Lake region of western People’s Republic of China, resulting in the death of ≈10,000 birds (*1**,**2*). On the basis of phylogenetic analysis of the hemagglutinin (HA) gene, the virus was classified as clade 2.2 according to the World Health Organization guidelines. Subsequently, viruses from this clade were found in Mongolia, Russia, Europe, and Africa along the migratory flyways of birds (*3**,**4*). This unique distribution of the same clade of HPAI virus (H5N1) through different migratory routes indicates that migratory birds might play a global role in virus dissemination (*3**,**4*).

In 2006, viruses from the same clade were isolated in the Qinghai Lake region (*3*). Analysis of viral outbreaks along migratory flyways demonstrated a similar outbreak pattern for the past 4 years (2006–2009) (*5*). During that period, clade 2.2 avian influenza virus (H5N1) was isolated in China, Mongolia, Russia, Germany, Egypt, and Nigeria; all viruses were closely related to the Qinghai Lake virus. Despite the broad distribution of clade 2.2 viruses in migratory flyways, few isolates of clade 2.2 viruses in local domestic poultry were reported, especially in China (*6*). Outbreaks of these viruses were reported in poultry in Africa (*7*). The reason these viruses rarely cause outbreaks in poultry is unknown.

During May–June 2009 and 2010, several dead migratory birds were found in the Qinghai Lake region. Nine HPAI viruses (H5N1) were isolated in 2009 and 2 were isolated in 2010 from great cormorants (*Phalacrocorax carbo*), brown-headed gulls (*Chroicocephalus brunnicephalus*), great black-headed gulls (*Ichthyaetus ichthyaetus*), great-crested grebes (*Podiceps cristatus*), and bar-headed geese (*Anser indicus*) and serotyped as described (*3*). HA genes from all 11 isolates were subsequently amplified by using reverse transcription–PCR and sequenced.

Phylogenetic analysis of HA sequences and an additional HA gene sequence from the 2009 Qinghai Lake subtype H5N1 virus isolate from a great crested grebe (from the National Avian Influenza Virus Reference Laboratory, Harbin, China) (GenBank accession no. CY063318) showed that HA genes from all 12 viruses clustered as clade 2.3.2 (Figure); none clustered with clade 2.2 viruses. Additionally, the HA cleavage site in the new isolates is PQRERRRKRG, which is identical to that of clade 2.3.2 viruses. In clade 2.2, the cleavage site is PQRERRRKKRG.

**Figure:**
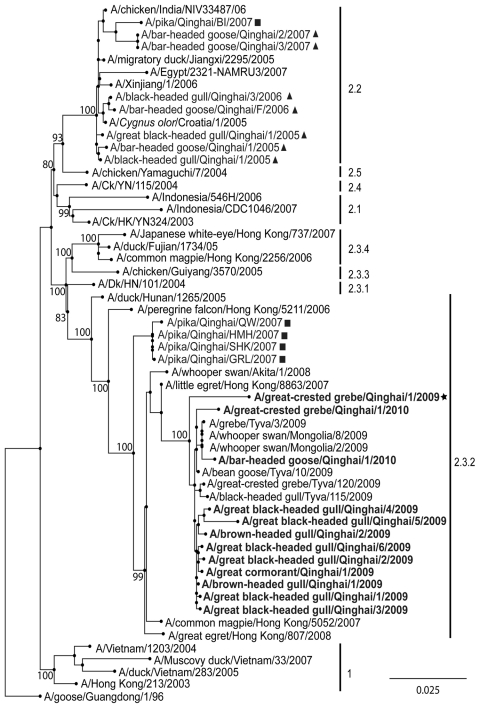
Bootstrapped (1,000×) maximum likelihood phylogenetic tree of hemagglutinin genes of avian influenza viruses (H5N1), People’s Republic of China, 2009–2010. Viruses isolated from the plateau pika near Qinghai Lake are indicated by squares; viruses isolated from wild birds in Qinghai Lake Region during 2005–2007 are indicated by triangles; 2009 Qinghai virus submitted to GenBank by the National Avian Influenza Virus Reference Laboratory (Harbin, China) is indicated by the star and in **boldface**; and viruses isolated in 2009 and 2010 in the Qinghai Lake Region are indicated in **boldface**, Clade numbers are indicated on the right. NAMRU3, Naval Medical Research Unit 3; Ck, chicken; Dk, duck. Scale bar indicates nucleotide substitutions per site.

A bootstrap (1,000×) maximum likelihood tree (*8*) also demonstrated that Qinghai 2009 and 2010 virus isolates are closely related to those isolated in Mongolia and Uvs Nuur Lake in 2009, as reported by Sharshov et al. (*5*). Qinghai Lake and Uvs Nuur Lake, which are found along the migratory flyway in central Asia, are major lakes for bird migration and breeding. Many birds fly from Qinghai Lake to Uvs Nuur Lake in the spring.

If one considers isolation date and bird species infected, viruses isolated in Mongolia and Russia and our isolates were likely transmitted between the 2 lake regions by bird migration. Moreover, HA sequences are closely related to viruses isolated from wild birds in Hong Kong and Japan during 2007–2008, which are the most recent isolates of clade 2.3.2 viruses before isolation of 2009 Qinghai Lake viruses. These results indicate that viruses in the Qinghai Lake region may be transmitted by wild birds along the migratory flyway in eastern Asia. However, there is no evidence that avian influenza virus (H5N1) is transmitted from eastern Asian (inner China or across the Himalayas) to the Qinghai Lake region.

The 2009 and 2010 Qinghai Lake viruses are related to various viruses isolated from plateau pikas near Qinghai Lake (*9*). In 2007, clade 2.2 and clade 2.3.2 viruses were isolated from plateau pikas, but no clade 2.3.2 viruses were found in aquatic birds. Wild birds, pikas, and other animals near Qinghai Lake share the same environment, and viruses may be transmitted across species. However, surveillance data are limited for wild animals near Qinghai Lake. Therefore, further investigations need to be conducted to clarify relationships among birds, animals, and influenza viruses near Qinghai Lake.

Our results and those of Sharshov et al. (*5*) show that in 2009 HPAI virus (H5N1) began infecting birds along the migratory route near Qinghai Lake and changed from clade 2.2 viruses to clade 2.3 viruses. New outbreaks of HPAI viruses (H5N1) along this migratory flyway should be investigated.
